# Surveillance of *Helicobacter pylori* Antibiotic Susceptibility in Indonesia: Different Resistance Types among Regions and with Novel Genetic Mutations

**DOI:** 10.1371/journal.pone.0166199

**Published:** 2016-12-01

**Authors:** Muhammad Miftahussurur, Ari Fahrial Syam, Iswan Abbas Nusi, Dadang Makmun, Langgeng Agung Waskito, Lukman Hakim Zein, Fardah Akil, Willy Brodus Uwan, David Simanjuntak, I Dewa Nyoman Wibawa, Jimmy Bradley Waleleng, Alexander Michael Joseph Saudale, Fauzi Yusuf, Syifa Mustika, Pangestu Adi, Ummi Maimunah, Hasan Maulahela, Yudith Annisa Ayu Rezkitha, Phawinee Subsomwong, Nasronudin, Dadik Rahardjo, Rumiko Suzuki, Junko Akada, Yoshio Yamaoka

**Affiliations:** 1 Department of Environmental and Preventive Medicine, Oita University Faculty of Medicine, Yufu, Japan; 2 Department of Medicine, Gastroenterology and Hepatology Section, Baylor College of Medicine, Houston, Texas, United States of America; 3 Division of Gastroentero-Hepatology, Department of Internal Medicine, Universitas Airlangga Faculty of Medicine, Surabaya, Indonesia; 4 Institute of Tropical Disease, Universitas Airlangga, Surabaya, Indonesia; 5 Division of Gastroenterology, Department of Internal Medicine, Faculty of Medicine, University of Indonesia, Jakarta, Indonesia; 6 Division of Gastroentero-Hepatology, Department of Internal Medicine, Faculty of Medicine, University of Sumatera Utara, Medan, Indonesia; 7 Center of Gastroentero-Hepatology, Department of Internal Medicine, Faculty of Medicine, Hasanuddin University, Makassar, Indonesia; 8 Department of Internal Medicine, Santo Antonius Hospital, Pontianak, Indonesia; 9 Department of Internal Medicine, Yowari Hospital, Jayapura, Indonesia; 10 Division of Gastroentero-hepatology, Department of Internal Medicine, Faculty of Medicine University of Udayana, Denpasar, Indonesia; 11 Division of Gastroentero-hepatology, Department of Internal Medicine, Faculty of Medicine, University of Sam Ratulangi, Prof. Dr. RD Kandou Hospital, Manado, Indonesia; 12 Department of Internal Medicine, Prof. Dr. W. Z. Johannes General Hospital, Kupang, Indonesia; 13 Division of Gastroentero-hepatology, Department of Internal Medicine, Dr. Zainoel Abidin General Hospital, Banda Aceh, Indonesia; 14 Division of Gastroentero-hepatology, Department of Internal Medicine, Dr. Saiful Anwar General Hospital, Malang, Indonesia; Universidad de Santiago de Compostela, SPAIN

## Abstract

Information regarding *Helicobacter pylori* antibiotic resistance in Indonesia was previously inadequate. We assessed antibiotic susceptibility for *H*. *pylori* in Indonesia, and determined the association between virulence genes or genetic mutations and antibiotic resistance. We recruited 849 dyspeptic patients who underwent endoscopy in 11 cities in Indonesia. E-test was used to determine the minimum inhibitory concentration of five antibiotics. PCR-based sequencing assessed mutations in *23S rRNA*, *rdxA*, *gyrA*, *gyrB*, and virulence genes. Next generation sequencing was used to obtain full-length sequences of *23S rRNA*, *infB*, and *rpl22*. We cultured 77 strains and identified 9.1% with clarithromycin resistance. Low prevalence was also found for amoxicillin and tetracycline resistance (5.2% and 2.6%, respectively). In contrast, high resistance rates to metronidazole (46.7%) and levofloxacin (31.2%) were demonstrated. Strains isolated from Sumatera Island had significantly higher metronidazole resistance than those from other locations. Metronidazole resistant strains had highly distributed *rdxA* amino acid substitutions and the *23S rRNA* A2143G mutation was associated with clarithromycin resistance (42.9%). However, one strain with the highest MIC value had a novel mutation in *rpl22* without an A2143G mutation. Mutation at Asn-87 and/or Asp-91 of *gyrA* was associated with levofloxacin-resistance and was related to *gyrB* mutations. In conclusions, although this is a pilot study for a larger survey, our current data show that Indonesian strains had the high prevalence of metronidazole and levofloxacin resistance with low prevalence of clarithromycin, amoxicillin, and tetracycline resistance. Nevertheless, clarithromycin- or metronidazole-based triple therapy should be administered with caution in some regions of Indonesia.

## Introduction

Asia is a very important continent for *Helicobacter pylori* infection, a common chronic bacterial infection in humans that is associated with peptic ulcer disease, gastric cancer, and primary gastric B-cell lymphoma [1]. Asia is the continent with the largest populace (4.4 billion people) and the highest frequency of gastric cancer in the world [2]. With some exceptions that some population with low incidence of gastric cancer even with a high prevalence of *H*. *pylori* known as the “Asian enigma” [3], the incidence of gastric cancer in several regions tends to mirror the prevalence of *H*. *pylori* infection [4]. Recent guidelines have proposed indications and regimens for the Asia-Pacific region and three countries in East Asia [5–8]. Nevertheless, the development of drug resistance in *H*. *pylori* is a major issue and the viability of some regimens has been truly challenged as they are becoming unsuccessful [9]. Creating an updated suitable first-line regimen is fundamental to counteract revised treatment courses that result in perpetuation of secondary antibiotic resistance [2].

Indonesia is a multi-ethnic nation in Southeast Asia that consists of more than 13,600 islands, with Sumatra, Papua, Kalimantan, Sulawesi, and Java being the five main islands. Currently, hospitals that provide GI endoscopy services in Indonesia are limited, and most are located on Java Island [10]. The prevalence of *H*. *pylori* infection in Javanese, the predominant ethnicity, is low (2.4%), even when using a combination of five different diagnostic methods [11,12]. However, several ethnic groups have much higher prevalence of *H*. *pylori* infection (42.9%, 40.0%, and 36.7% for Papuan, Batak, and Buginese individuals, respectively) [12]. Additionally, they harbor more virulent genotypes such as *cagA* positive, *oipA* ‘on’, and *iceA1* positive [13]. The Kyoto Global Consensus Conference on *H*. *pylori* Gastritis in 2014 expressed that *H*. *pylori* gastritis should be characterized as an infectious disease, notwithstanding asymptomatic patients and irrespective of complications such as peptic ulcers and gastric cancer [14]. It has been proposed that *H*. *pylori* should be eradicated, even in Indonesia. To our knowledge, only a single study has reported the rates of *H*. *pylori* antibiotic resistance in Indonesia, identifying 72 strains in the low prevalence *H*. *pylori* region, Jakarta, in 2006 [15]. In this study, the rates clarithromycin (CAM), amoxicillin (AMX), metronidazole (MNZ), and levofloxacin (LVX) resistance were 27.8%, 19.4%, 100.0%, and 1.4%, respectively [15]. In addition, there was no report regarding tetracycline (TCN) resistance in Indonesian in *H*. *pylori* strains. TCN is a basic antibiotic, used in quadruple regimens, for *H*. *pylori* eradication. Since antibiotic resistance is expanding globally [16,17], it is necessary to examine the drug resistance rates in Indonesia.

The understanding of *H*. *pylori* antibiotic resistance mechanisms, which primarily occur because of mutations in chromosomal genes, is important as a premise for the establishment of rational antibiotic combinations. Critically, although numerous point mutations have appeared, the positions of such mutations were not uniform for every topographical area [18,19]. For example, although two nucleotide substitutions, specifically, A2142G or A2142C and A2143G in the peptidyl transferase loop of *23S rRNA*, cause primary CAM resistance in *H*. *pylori* strains isolated in Western countries [20], they account for only 23% of resistant strains in Asia [20]. Our previous report demonstrated the synergic effect of mutated sequences in *hp1048 (infB*), *hp1314 (rpl22)*, and A2143G, which resulted in higher MICs [21]. Unlike the mutational patterns of *23S rRNA*, the mechanisms of MNZ resistance are complex and largely associated with inactivating mutations in *rdxA*, through frameshift mutations, insertions, and deletions [22]. Moreover mutations in the sequences of gyrase subunit A (*gyrA*) and *gyrB*, encoding translational proteins, greatly reduce the antimicrobial ability of fluoroquinolones [23].

In this study, we aimed to determine the antibiotic susceptibility of *H*. *pylori* in 11 cities, covering the five largest islands, of Indonesia. We also analyzed the association between virulence genes and antibiotic resistance rates. Furthermore, we determined the presence of genetic mutations that are associated with antibiotic resistance.

## Materials and Methods

### Patients and *H*. *pylori*

We conducted a prospective study from August 2012 to November 2015. This study included 849 adult dyspeptic patients who underwent endoscopy examinations in 11 cities including cities on the five largest islands of Indonesia. Patients with bleeding related to esophageal varices, a history of partial gastric resection and previous *H*. *pylori* eradication therapy were excluded from this study. Included were patients from Surabaya (296 patients), Jakarta (31), and Malang (97) on Java Island, Aceh (38) and Medan (93) on Sumatera Island, Pontianak (90) on Kalimantan Island, Makassar (30) and Manado (57) on Sulawesi Island, Jayapura (21) on Papua Island, Bangli (61) on Bali Island, and Kupang (35) on Timor Island. Peptic ulcer diseases were diagnosed by endoscopic observation, whereas chronic gastritis was determined by histologic examination. All procedures contributing to this work comply with the ethical standards of the relevant national and institutional committees on human experimentation and with the Helsinki Declaration of 1975, as revised in 2008. There were no minors or children enrolled in our study, therefore not needed informed consent from the next of kin, caretakers, or guardians on behalf of them. Written informed consent was obtained from all participants, and the study protocol was approved by the Institutional Review Board or the Ethics Committee of Dr. Cipto Mangunkusumo Teaching Hospital (Jakarta, Indonesia), Dr. Soetomo Teaching Hospital (Surabaya, Indonesia), Dr. Wahidin Sudirohusodo Teaching Hospital (Makassar, Indonesia), and Oita University Faculty of Medicine (Yufu, Japan).

For *H*. *pylori* culture, antral biopsy specimens were homogenized and inoculated onto antibiotics selection plate, subsequently subcultured onto Mueller Hinton II Agar medium (Becton Dickinson, NJ, USA) supplemented with 10% horse blood without antibiotics. The plates were incubated for up to 10 days at 37°C under microaerophilic conditions (10% O_2_, 5% CO_2_, and 85% N_2_). *H*. *pylori* isolates were identified based on colony morphology, Gram staining results, and positive reactions for oxidase, catalase, and urease. Isolated strains were stored at -80°C in Brucella Broth (Difco, NJ, USA) containing 10% dimethyl sulfoxide and 10% horse serum.

### Antibiotic susceptibility testing

The E-test method (Biomerieux, France) was used to determine the minimum inhibitory concentration (MIC) of AMX, MNZ, TCN, CAM, and LVX. Mueller-Hinton II Agar medium (Becton Dickinson) supplemented with 10% defibrinated horse blood was used as culture media. The bacterial suspension, adjusted to be equivalent to a McFarland opacity standard of 3.0, was inoculated onto the plates. After 72 h of incubation, the MIC of each antibiotic was determined. Quality control was performed using *H*. *pylori* ATCC 43504. The resistance breakpoints were determined as described by the European Committee on Antimicrobial Susceptibility Testing (EUCAST; available at http://www.eucast.org/). Strains were considered to be resistant for MICs of >0.125 mg/L for AMX, 0.25 mg/L for CAM, 8 mg/L for MNZ, and 1 mg/L for TCN and LVX.

### Virulence factors and resistant strains molecular detection

*H*. *pylori* DNA was extracted from *H*. *pylori*, cultured to confluence on plates, using a commercially available kit (Qiagen, Hilden, Germany). The DNA was stored at -20°C until used for gene amplification. Mutations in *gyrA*, *gyrB*, *rdxA*, and *23S rRNA* were assessed in antibiotic-resistant strains by PCR-based sequencing using the primers as described previously [24–26]. As a control, we sequenced five randomly selected strains sensitive to MNZ and LVX and one CAM-sensitive strain from the collection of Indonesian strains. The sequences were then compared with the published sequence of *H*. *pylori* strain 26695 (GenBank accession number AE000511.1 GI: 6626253) using MAFFT version 7 (available at http://mafft.cbrc.jp/alignment/server/) and confirmed by visual inspection. Direct sequencing of the *cagA* EPIYA repeat region and determination of *oipA* status (“on” or “off”) were determined by PCR-based sequencing as described previously [27–29]. The presence of *vacA* (s1 or s2, m1 or m2 and i1, i2 or i3), *iceA* (*iceA1* or *iceA2*), *dupA*, *jhp0562*, and *β-(1*,*3)galT* genotypes were determined based on PCR product size as described previously [30–35].

To find other genetic mutations, associated with high MIC values, but not involving typical *23S rRNA* mutations, we also obtained full-length *23S rRNA*, *hp1048* (*infB*), and *hp1314* (*rpl22*) [21] from next-generation sequencing data (MiSeq next-generation sequencer; Illumina, Inc., San Diego, CA). MiSeq output was integrated into contig sequences by CLC Genomics Workbench 7.0.4. Genomics Workbench was also used for gene prediction and translation to protein sequences.

### Statistical analysis

Discrete variables were tested using the chi-square test, whereas continuous variables were tested using the Mann-Whitney *U* and *t*-tests. A multivariate logistic regression model was used to calculate the odds ratios (OR) of clinical outcomes including age, sex, *H*. *pylori* antibiotic resistance, ethnicity, and location. All determinants with P values < 0.10 were entered together into the full logistic regression model. The OR and 95% confidence interval (CI) were used to estimate risk. A P value of < 0.05 was accepted as statistically significant. The SPSS statistical software package version 18.0 (SPSS, Inc., Chicago, IL) was used for all statistical analyses.

## Results

### Prevalence of antibiotic resistance

Despite obtaining a large amount of samples, only 77 strains could be isolated. The low number of isolated strains was related to the low prevalence of *H*. *pylori* infection in Indonesia, and was confirmed by histology and immunohistochemistry (88/849, 10.4%, Table 1). Half of the samples were obtained from Java Island (424 samples) and had a *H*. *pylori* infection prevalence of only 4.0% (17/424; Table 1). In contrast, the most recent survey of Timor Island found the prevalence of *H*. *pylori* infection to be 40.0% (14/35). The host individuals consisted of 39 males (age range, 24 to 80 years; mean age, 49.6 ± 10.8 years) and 38 female patients (age range, 28 to 68 years; mean age 48.9 ± 15.4 years). Strains were isolated from Surabaya (12 strains), Jakarta (1), Malang (1), Medan (18), Pontianak (5), Makassar (6), Manado (7), Jayapura (7), Bali (6), and Kupang (14 strains). Among the patients, 70 had chronic gastritis and seven had peptic ulcer diseases.

**Table 1:** Prevalence of *H*. *pylori* infection in Indonesia based on multiple tests.

Island (city)	Year	N	Diagnostic Method (%)		
			Culture	Histology confirmed by IHC	At least one method
Bali (Bangli)*	2015	61	6 (9.8)	7 (11.5)	7 (11.5)
Java		424	14 (3.3)	15 (3.5)***	17 (4.0)
(Surabaya)	2012–2015	296	12 (4.1)	14 (4.7)	15 (5.1)
(Jakarta)	2013	31	1 (0.1)	1 (0.1)	1 (0.1)
(Malang)*	2014	97	1 (1.0)	**	1 (1.0)
Kalimantan (Pontianak)	2014	90	5 (5.6)	4 (4.4)	6 (6.7)
Papua (Jayapura)	2013	21	9 (42.9)	9 (42.9)	9 (42.9)
Sumatera		131	19 (14.5)	20 (15.3)	26 (19.8)
(Medan)	2014	93	19 (20.4)	20 (21.5)	26 (27.9)
(Aceh)*	2014	38	0 (0.0)	0 (0.0)	0 (0.0)
Sulawesi		87	13 (14.9)	13 (14.9)	13(14.9)
(Manado)*	2015	57	7 (12.3)	7 (12.3)	7 (12.3)
(Makassar)	2014	30	6 (20.0)	6 (20.0)	6 (20.0)
Timor (Kupang)*	2015	35	14 (40.0)	12 (34.3)	14 (40.0)
Total		849	80 (9.4)	80 (9.4)***	88 (10.4)

IHC: Immunohistochemistry

* The most recent surveys that are not including in the previous publication (Syam AF, *et al*., 2015)

** Sample obtained only from culture, there was no sample for histology examination

*** The total number does not include the Malang survey

**Fig 1:**
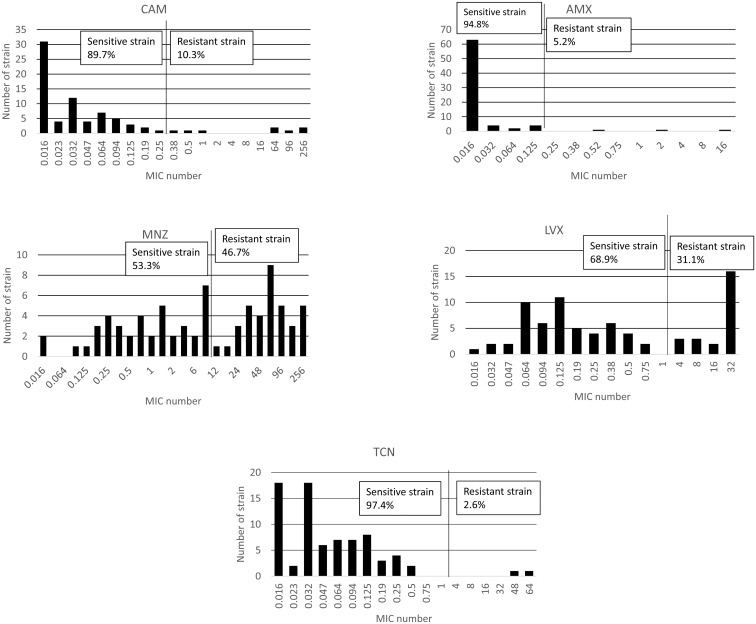
Distribution of antibiotic MIC values in Indonesia. The resistance rates to metronidazole and levofloxacin were high; in contrast, we revealed a low prevalence of clarithromycin, amoxicillin, and tetracycline resistance.

Overall, there were 28 strains that were sensitive to all antibiotics (36.4%). In contrast with the previous study in Indonesia [15], we found a low prevalence of CAM resistance (7/77, 9.1%, compared to 27.8% in the previous study). Low prevalence was also observed for AMX resistance (4/77, 5.2%) and TCN resistance (2/77, 2.6%, Table 2). In contrast, and in accordance with the trend of increasing resistance Asia [36], there was high rate of resistance to MNZ (36/77, 46.8%). Moreover, most strains were associated with MIC values ≥ 48 mg/L (26/36, 72.2%, Fig 1). In addition, we detected a high prevalence of LVX resistance (24/77, 31.2%) with a high distribution of MIC values that were predominantly ≥ 16 mg/L. The distribution of patient age and antimicrobial resistance of the isolates is shown in Table 2. Antibiotic resistance rate did not differ among different age groups (P = 0.37, 0.42, 0.07, 0.56, and 0.45 for AMX, CAM, MNZ, LVX, and TCN, respectively). There were no associations between antibiotic resistance and gender or clinical outcomes (P >0.05).

**Table 2:** Prevalence of antibiotic resistance of *H*. *pylori* isolates in Indonesia.

Characteristic	N	Antibiotics (%)				
		CAM	AMX	MNZ	LVX	TCN
Total	77	7 (9.1)	4 (5.2)	36 (46.7)	24 (31.2)	2 (2.6)
Gender						
Male	38	3 (7.9)	2 (5.2)	21 (55.2)	11 (28.9)	1 (2.7)
Female	39	4 (10.2)	2 (5.1)	15 (38.4)	13 (33.3)	1 (2.6)
Age Groups						
17–30	9	0 (0.0)	0 (0.0)	6 (66.6)	3 (33.3)	0 (0.0)
31–40	10	2 (20.0)	0 (0.0)	6 (60.0)	3 (30.0)	1 (10.0)
41–50	21	2 (9.1)	1 (4.7)	5 (22.7)	4 (18.1)	1 (4.5)
51–60	25	1 (4.1)	1 (4.0)	14 (58.3)	10 (41.6)	0 (0.0)
>61	12	2 (16.6)	2 (16.6)	5 (41.6)	4 (33.3)	0 (0.0)
Clinical Outcome						
Gastritis	70	6 (8.9)	4 (5.9)	30 (44.7)	21 (31.3)	0 (0.0)
PUD	7	0 (0.0)	0 (0.0)	3 (42.8)	0 (0.0)	0 (0.0)

Abbreviations: AMX, amoxicillin; CAM, clarithromycin; MNZ, metronidazole; TCN, tetracycline; LVX, levofloxacin; PUD, peptic ulcer disease

### Antibiotic resistance rates according to location

The prevalence of antibiotic resistance based on location is shown in Table 3. Strains from Kalimantan were resistant to only two antibiotics. Strains obtained from Java and Bali Islands had CAM resistance rates greater than 15%, which is the permitted limit of CAM resistance recommended to be used for eradication therapy without checking the resistant rate [37,38]. AMX resistance was not detected in strains obtained from three islands (Bali, Java, and Kalimantan). Only Java Island showed signs of increased resistance to TCN (2/14, 14.3%). The highest LVX resistance was in strains isolated from Java and Sumatera Islands (50.0% and 44.4%, respectively). Strains isolated from Sumatera Island showed significantly higher MNZ resistance (88.9%) than those of other locations (50.0%, 42.9%, 33.3%, 30.8%, 21.4% and 20.0% for Java, Papua, Bali, Sulawesi, Timor, and Kalimantan, respectively, P = 0.003). Even after adjustment by age and sex in multivariate analysis, strains from Sumatera Island had significantly higher MNZ resistance rates than strains from other islands (OR = 7.9, OR = 10.5, OR = 15.3, OR = 28.3, OR = 17.5, and OR = 30.4 for Java, Papua, Bali, Sulawesi, Timor, and Kalimantan Island, respectively, P <0.05).

**Table 3:** Prevalence of *H*. *pylori* antibiotic resistance in Indonesia by location.

Island	N	Resistance (%)				
		CAM	AMX	MNZ*	LVX	TCN
Bali	6	1 (16.7)	0 (0.0)	2 (33.3)	1 (16.6)	0 (0.0)
Java	14	3 (21.4)	0 (0.0)	7 (50.0)	7 (50.0)	2 (14.3)
Kalimantan	5	0 (0.0)	0 (0.0)	1 (20.0)	1 (20.0)	0 (0.0)
Papua	7	1 (14.3)	1 (14.3)	3 (42.9)	2 (28.6)	0 (0.0)
Sulawesi	13	1 (7.7)	1 (7.7)	4 (30.8)	2 (15.4)	0 (0.0)
Sumatera	18	1 (5.6)	1 (5.6)	16 (88.9)	8 (44.4)	0 (0.0)
Timor	14	0 (0.0)	1 (7.1)	3 (21.4)	3 (21.4)	0 (0.0)

Abbreviations: AMX, amoxicillin; CAM, clarithromycin; MNZ, metronidazole; TCN, tetracycline; LVX, levofloxacin.

* Strains isolated from Sumatera Island had significantly higher MNZ resistance rates than strains isolated from other islands even after adjusting for age and sex (P <0.05).

### Antibiotic resistance rate according to ethnic group

The predominant ethnic groups from different islands and their association with antibiotic resistance rates are shown in Table 4. Strains isolated from Ambonese, Chinese, and Balinese individuals displayed a high prevalence of CAM resistance (50.0%, 20.0%, and 16.6%, respectively, Table 4).

**Table 4:** Prevalence of antibiotic resistance in *H*. *pylori* in Indonesia by ethnicity.

Ethnicity	Island	N	Resistance (%)				
			CAM	AMX	MNZ	LVX	TCN
Javanese	Java	3	0 (0.0)	0 (0.0)	1 (33.3)	1 (33.3)	0 (0.0)
Chinese**	Java and Kalimantan	10	2 (20.0)	0 (0.0)	5 (50.0)	5 (50.0)	1 (10.0)*
Batak	Sumatera	19	1 (5.2)	1 (5.2)	16 (84.2)*	8 (42.1)	0 (0.0)
Papuan	Papua	7	1 (14.3)	1 (14.3)	3 (42.9)	2 (28.6)	0 (0.0)
Dayak	Kalimantan	2	0 (0.0)	0 (0.0)	0 (0.0)	0 (0.0)	0 (0.0)
Buginese	Sulawesi	6	0 (0.0)	1 (16.6)	2 (33.3)	0 (0.0)	0 (0.0)
Balinese	Bali	6	1 (16.6)	0 (0.0)	2 (33.3)	1 (16.6)	0 (0.0)
Timor	Timor	15	0 (0.0)	1 (6.6)	3 (20.0)	4 (26.6)	0 (0.0)
Minahasanese	Sulawesi	7	1 (14.3)	0 (0.0)	2 (28.6)	2 (28.6)	0 (0.0)
Ambonese***	Java	2	1 (50.0)	0 (0.0)	2 (100.0)*	1 (50.0)	1 (50.0)*

Abbreviations: AMX, amoxicillin; CAM, clarithromycin; MNZ, metronidazole; TCN, tetracycline; LVX, levofloxacin.

* P < 0.05

** Chinese-Indonesians are dispersing throughout the archipelago. In this study, the strains were obtained from Chinese individuals who lived in Surabaya, Java Island, Pontianak, and Kalimantan Island.

*** Ambonese are the predominant group of Ambon Island in Maluku, an island group east of Sulawesi. In this study, the strains were obtained from Ambonese who lived in Surabaya, Java Island.

Only those from Buginese individuals showed AMX resistance >15%. Among three ethnicities with the highest prevalence of *H*. *pylori* in Indonesia [12], Papuan and Batak individuals were associated with a large number of antibiotic resistance types, including LVX, which is part of a second-line regimen, in contrast to strains isolated from Buginese individuals. Ambonese individuals were associated with higher rates of MNZ and TCN resistance than other ethnicities (both P = 0.01). Only strains from Dayak individuals were sensitive to all five antibiotics.

### Multidrug resistance

No strain was resistant to all tested antibiotics (Table 5). Fifteen strains showed dual-drug resistance (11 strains for MNZ-LVX, two for CAM-LVX and one each for MNZ-AMX and LVX-AMX). Resistance to three antibiotics was observed in two (2.6%) strains that were isolated from Java Island; one was resistant to a combination of CAM, MNZ, and LVX. In addition, two strains (obtained from Sumatera and Java Island) were recognized as resistant to four drugs including LVX. Overall, Java (six strains) and Sumatera Island (seven strains) showed a higher prevalence of multidrug resistance than other locations. No differences were observed for clinical outcomes between single-drug and multidrug resistant infections (P = 0.53).

**Table 5:** Prevalence of multidrug resistance among Indonesian strains.

Antibiotics	Total	Number of Patients (%)						
		Bali N = 6	Kalimantan N = 5	Java N = 14	Papua N = 7	Sulawesi N = 13	Sumatera N = 18	Timor N = 14
Double drugs								
MNZ + AMX	1	0 (0.0)	0 (0.0)	0 (0.0)	0 (0.0)	1 (7.7)	0 (0.0)	0 (0.0)
MNZ + LVX	11	1 (16.7)	0 (0.0)	2 (14.3)	1 (14.3)	0 (0.0)	6 (33.3)	1 (7.1)
CAM + LVX	2	0 (0.0)	0 (0.0)	1 (7.1)	0 (0.0)	1 (7.7)	0 (0.0)	0 (0.0)
LVX + AMX	1	0 (0.0)	0 (0.0)	0 (0.0)	1 (14.3)	0 (0.0)	0 (0.0)	0 (0.0)
Triple drugs								
CAM + MNZ + LVX	1	0 (0.0)	0 (0.0)	1 (7.1)	0 (0.0)	0 (0.0)	0 (0.0)	0 (0.0)
TCN + MNZ + LVX	1	0 (0.0)	0 (0.0)	1 (7.1)	0 (0.0)	0 (0.0)	0 (0.0)	0 (0.0)
Quadruple drugs								
CAM + MNZ + AMX + LVX	1	0 (0.0)	0 (0.0)	0 (0.0)	0 (0.0)	0 (0.0)	1 (5.6)	0 (0.0)
CAM + MNZ + TCN + LVX	1	0 (0.0)	0 (0.0)	1 (7.1)	0 (0.0)	0 (0.0)	0 (0.0)	0 (0.0)

Abbreviations: AMX, amoxicillin; CAM, clarithromycin; MNZ, metronidazole; TCN, tetracycline; LVX, levofloxacin.

### Virulence genes of Indonesian strains and antibiotic resistance types

Table 6 shows the association between virulence genes and the pattern of antibiotic resistance. Although there was a significant association between TCN resistance and *cagA* positivity (P = 0.004), it was only 2 resistant strains which were insufficient to make conclusion. *jhp0562*-positive/*β-(1*,*3)galT*-negative *H*. *pylori* types tended to have higher MNZ resistance than *jhp0562*-negative/*β-(1*,*3)galT*-positive types (P = 0.06). There was no association between other virulence factors and antibiotics resistance types (P >0.05).

**Table 6:** Association between virulence genes and antibiotic resistance pattern (%).

Virulence Genes	CAM (%)		MNZ (%)		LVX (%)	
	S	R	S	R	S	R
Strain (number)	70	7	41	36	53	24
*cagA* positive	94.3	100	95.1	94.4	96.2	91.7
*cagA* type						
• East-Asian-type	60.0	85.7	53.7	72.2	58.5	70.8
• Western-type	25.7	0.0	29.3	16.7	28.3	12.5
• ABB-type	8.6	14.3	12.1	5.6	9.4	8.3
*vacA* s1/m1	65.7	85.7	75.6	58.3	67.9	66.7
*iceA1* genotype	64.3	71.4	61.0	69.4	67.9	58.3
*jhp0562* genotype	20.0	0.0	24.4	11.1	22.6	8.3*
*oipA* “on”	91.4	100.0	90.2	94.4	92.5	91.7
*dupA* negative	82.9	100	80.5	88.9	88.7	75.0

* P = 0.06The data about the tetracycline and amoxicillin were excluded from the table, for which the resistant strains were 5 or less.

Abbreviations: AMX, amoxicillin; CAM, clarithromycin; MNZ, metronidazole; TCN, tetracycline; LVX, levofloxacin; *iceA1* genotype, *iceA1* positive/ *iceA2* negative; *jhp0562* genotype, *jhp0562* positive/ *β-(1*,*3)galT* negative.

### Detection of *H*. *pylori* gene mutations and association with antimicrobial resistance

Two MNZ-resistant strains did not show identifiable specific bands for *rdxA* after targeted PCR; therefore, 34 MNZ-resistant and 5-sensitive control Indonesian strains were analyzed in this study. DNA sequence analysis of *rdxA* from all MNZ-sensitive strains revealed intact reading frames (lacking nonsense mutations). Pairwise alignment identified that the MNZ-sensitive strains shared 94.9–97.0% identity with the reference strain, 26695. In contrast, most MNZ-resistant strains contained missense mutations (16/34, 47.1%), resulting in amino acid substitutions (Table 7). In addition, five strains had nonsense mutations that resulted in the introduction of a premature stop codon. Moreover, the *rdxA* alleles of nine strains (26.5%) contained nucleotide deletions and/or insertions that resulted in a translational frameshift.

**Table 7:** Mutation type of *rdxA* related to metronidazole resistance.

No	Strain	MIC	Sites of Mutation	Type of mutation
1	Jayapura16	32	R16C, A183V	Missense mutation
2	Kupang23	>256	E15Q, R16H, E32D, E34stop	Premature stop codon
3	Kupang 30	96	R90K, P106S, V111A	Missense mutation
4	Kupang 41	64	Q6H, R16C, R90K, E175Q, N178D, A183V	Missense mutation
5	Kupang 5	12	A68V	Missense mutation
6	Kupang 73	48	K64N, H97T, P106S, H127Y, Q197stop	Premature stop codon
7	Malang1	48	16frameshift	Frameshift mutation
8	Manado26	64	None	Non-specific mutation
9	Manado 31	48	65frameshift	Frameshift mutation
10	Medan3	16	65frameshift	Frameshift mutation
11	Medan10	>256	H97Y, P106T, G122S	Missense mutation
12	Medan15	64	P51S, A68T, E175Q, R176H	Missense mutation
13	Medan17	128	R16C, A68T, G122S, C159Y	Missense mutation
14	Medan18	64	R90K, 195frameshift	Frameshift mutation
15	Medan19	128	Q6H, R16C, R90K, H97T, A118S, E175Q, A183V, V204I	Missense mutation
16	Medan20	64	K64N, H97Y, P106T, G122S, 137frameshift	Frameshift mutation
17	Medan22	32	R16H, K64N, H97Y, P106M, G122S, E175Q	Missense mutation
18	Medan23	24	V55M, K64N, G122S, E175Q, A183V,	Missense mutation
19	Medan25	64	M1V, R16C, K64N, A80T, H97Y, P106T, G122S, E175Q	Missense mutation
20	Medan27	>256	4frameshift	Frameshift mutation
21	Medan28	48	P51S, K64N, M102stop, P106L, 173frameshift	Frameshift mutation
22	Medan30	96	None	Non-specific mutation
23	Medan31	96	K64N, H97I, P106T, G122S, E175stop	Premature stop codon
24	Medan32	32	R10I, A68V, G145R	Missense mutation
25	Medan33	96	R10S, H97Y, P106T, G122S, 169frameshift	Frameshift mutation
26	Makassar52	64	None	Non-specific mutation
27	Makassar55	96	Q6H, R16H, M56I, R90K, H97Y, P166S,	Missense mutation
28	Pontianak20	>256	G163D, E173stop	Premature stop codon
29	Surabaya68	24	R16H, P106T, V204I	Missense mutation
30	Surabaya69	32	None	Non-specific mutation
31	Surabaya79	128	Q6H, R16C, M56I, A80T, R90K, H97Y	Missense mutation
32	Surabaya137	>256	23frameshift	Frameshift mutation
33	Surabaya283	32	Q146stop	Premature stop codon
34	Surabaya304	64	I182V	Missense mutation

R16C means Cysteine replaced Arginine amino acid in the position 16; E34stop means stop codon replaced Glutamate amino acid in the position 34; 16frameshift means frameshift mutation in the position 16.

Among the five LVX-sensitive control Indonesian strains, no mutations in both *gyrA* and *gyrB* subunits were identified. In contrast, among 24 LVX-resistant strains, 22 had amino acid variants of the *gyrA* subunit (Table 8). Eleven LVX-resistant strains (45.8%) had an amino acid substitution at Asp-91, whereas six strains had an amino acid substitution at Asn-87. Both of these mutations (13/15, 86.7%) were predominantly associated with the highest MIC values observed (32 mg/L). In addition, two strains exhibited amino acid substitutions at Arg-484 and at Ser-479 in the *gyrB* subunit. When analyzing the association between these two genes and LVX-resistance, it was demonstrated the four strains (16.7%) had mutations in both *gyrA* and *gyrB*; 18/24 (75.0%) in *gyrA* only, and no strains had a mutation in *gyrB* only. We also found two of 24 (8.3%) LVX-resistant strains with no *gyrA* and *gyrB* mutations, which were associated with MIC values of 8 and 25 mg/L. There was no correlation between the degree of LVX resistance and the types or number of mutations in both genes.

**Table 8:** Mutation type of *gyrA* and *gyrB* related to levofloxacin resistance.

No	Strain	MIC (mg/L)	*gyrA* mutation	*gyrB* mutation
1	Jayapura1	>32	N87K	None
2	Jayapura21	>32	N87K	None
3	Kupang2	4	D91N, A129T	S479G
4	Kupang11	>32	D91Y	None
5	Kupang23	>32	A129T	S479G
6	Kupang41	8	D91N	R484K
7	Malang1	16	D91N	None
8	Manado18	8	None	None
9	Manado20	8	D91Y	None
10	Medan3	>32	N87I	None
11	Medan 10	25	None	None
12	Medan15	>32	R140K, D192N	None
13	Medan 17	16	D34N	None
14	Medan18	4	D91G, D161N	None
15	Medan22	>32	D91N	None
16	Medan 23	4	D34Y, R140K	None
17	Medan30	>32	D91N	None
18	Pontianak50	>32	D91G	None
19	Surabaya71	>32	D91N	None
20	Surabaya79	>32	N87Y	R484K
21	Surabaya137	>32	N87K	None
22	Surabaya151	>32	N87K	None
23	Surabaya283	>32	D91Y	None
24	Surabaya304	>32	D91G	None

N87K means Lysine replaced Asparagine amino acid in the position 87.

Based on *23Sr RNA* sequencing in the seven CAM-resistant strains, three (42.9%) exhibited an interesting point mutation, specifically A2143G (Table 9), and two of these strains were associated with high MIC values (64 and 96 mg/L). In contrast, we found minimal nucleotide variation on the one control Indonesian CAM-sensitive strain. Interestingly, there was no specific mutation in the strain associated with the highest MIC values (Surabaya137). Based on the previous report [21], we performed next generation sequencing of the Surabaya137 strain (average sequencing depth was 46.9× and overall %GC was 38.4). Using strain 26695 and the control CAM-sensitive strain Medan27, we could not identify any mutations in full-length *23S rRNA*. Although we found point mutations, (T189C and T198C) in *hp1048* (*infB*), these were silent mutations. In contrast, compared to strains 29965 and Medan27, we confirmed the involvement of novel mutated sequences in *hp1314* (*rpl22*) including 19 bp deletions at position 535 (Table 9).

**Table 9:** Gene mutations related to clarithromycin resistance.

No	Strain	MIC (mg/L)	*23SrRNA*	*rpl22 (hp1314)*	*infB (hp1048)*
1	Jayapura6	96	A2143G	-	-
2	Kupang64	32	None	-	-
3	Manado20	64	None	-	-
4	Medan15	0.5	None	-	-
5	Surabaya71	64	A2143G	-	-
6	Surabaya137	>256	None	None	A352G, C361T, G374A, A391G, C406A, T437C, G529A, C634D, G635T, G671A, T672C, A673T, 535del, T946C, G995T, G2245A
7	Surabaya304	1	A2143G, G2172T	-	-

A2143G means Guanine replaced Adenosine in the position 2143.

### Nucleotide sequencing

Nucleotide sequence data reported are available under the DDBJ accession numbers LC174777-LC174814 and LC175851 (*rdxA*), LC174815-LC174843 (*gyrB*), LC174844-LC174852 and LC174855-LC174874 (*gyrA*), LC175228-LC175232 and LC175234-LC175236 (*23srRNA*), LC175237-LC175238 (*infB*), LC175239-LC175240 (*rpl22*), LC174875-LC174908 (*cagA*) and LC175814-LC175850 (*oipA*).

## Discussion

Southeast Asia is a region with low CAM resistance rates [2]. The previous studies reported that CAM resistance rates among *H*. *pylori* isolates of Thailand and Malaysia, neighboring countries, were very low (3.7% and 5.2%, respectively) [18,39]. In agreement with this data, we revealed a low prevalence of CAM resistance. It is suggested that CAM-based triple therapy might still be useful as an initial treatment of *H*. *pylori* infection in Indonesia. However, further analysis based on the ethnicity of the host revealed that Ambonese, Chinese, and Balinese individuals were associated with strains with CAM resistance rates, exceeding those of the recommendations from the Maastricht guidelines (>15–20%) [37,38]. Furthermore, breakdown based on location showed that strains isolated from Bali and Java Island demonstrated high CAM resistance. Our report was consistent with a previous study in Indonesia, in which isolates from Java Island had a CAM resistance rate of 27.8% [15]. A recent report from Singapore, a neighboring country, also demonstrated a changing CAM-resistance profile over 15 years (7.9–17.1%) [40]. Therefore, triple therapy with CAM should be utilized with caution or should be culture-based in some areas, and involving particular ethnicities, in Indonesia.

We identified a mononucleotide substitution from A to G at site 2143 in CAM-resistant strains. Moreover, this was associated with high CAM MIC values (strains Jayapura6 and Surabaya304). A previous study reported the A2143G mutation has a much stronger impact on CAM resistance than the A2142G and A2142C mutations [41]. However, we failed to identify T2183C and A2223G mutations, which are frequently found to be the cause of CAM resistance in Asian, rather than in Western, countries [19]. Our current study also confirmed that *hp1314* (*rpl22*) mutations are associated with CAM resistance [21], although we identified different mutation types compared to those of Vietnamese strains (3 bp deletion and 9 bp insertion in *rpl22*). In contrast to the current study, we previously demonstrated that a single mutation in *infB* or *rpl22* resulted in low MIC values and showed the involvement of *infB*, *rpl22*, and *23S rRNA* in higher MIC values. Therefore, we suggested that *rpl22* mutations might not only result in synergistic effects, but also could be independent causes of CAM resistance.

Aside from isolates from Buginese individuals, for which the AMX resistance rate was greater than 15%, in general, our study revealed that Indonesian isolates had low resistance to AMX. We found only one resistant strain each from four islands. This is in contrast to the previous report in Indonesia [15], but similar to other typical Southeast Asian antibiotic resistance patterns [2]. This distinction might be based on differences in research facility reproducibility, caused by a lack of standardized testing protocols or different regional practices. Thus, AMX might be useful as a secondary antibiotic, for use in cases exhibiting poor responses to CAM-based triple therapy in Indonesia. This drug has been the first antibiotic utilized for *H*. *pylori* treatment because of the assumed absence of resistance [42]. Nevertheless, increasing primary AMX-resistance rates have been reported in South Korea (7.1–18.5%) [43], and in India and Pakistan (72.5% and 37.0%, respectively) [44,45]. Additionally, AMX can be obtained without prescription and has been one of the most commonly used antibiotics in Indonesia in recent years. A strict policy for antibiotic use is necessary to counteract the failure of primary antibiotic treatment for *H*. *pylori* in Indonesia.

TCN is an anti-microbial, to which resistance is occasionally experienced. The reason for this is that to achieve resistance, three point changes are required [9]. In agreement with most countries [46], we detected low TCN resistance in Indonesian strains. Although Ambonese individuals were associated with significantly higher resistance than strains isolated from other locations, the observation of only two resistant strains is of some concern. This is the first study to examine the TCN resistance rate in Indonesia. Due to the important role of TCN as a salvage quadruple therapy, our data are vital for guiding second line regimens for eliminating *H*. *pylori* infections.

The resistance rate for MNZ was high in Indonesia. Moreover, two *H*. *pylori* groups, specifically, Batak and Papua, had rates higher than the preferential number outlined by the Maastricht III Consensus Report (>40%) [37]. However, in Papua, the number of strains was small. In Asia, only Japan, Thailand, and Malaysia have populations associated with <40% MNZ resistance [2]. Therefore, regimens including MNZ are not suitable and should not be chosen as first-line treatments in Indonesia. We distinguished diverse mutations involving *rdxA* in most MNZ-resistant strains. Complex genetic events (insertions, deletions, and missense and frameshift mutations) were simultaneously present in the strains. Previously it was shown that only 11.8% of MNZ-resistant strains did not harbor any mutation in *rdxA*, and this was probably related to *frxA* [22], *rpsU* [47], *dppA* or *dapF* [48] alterations. Conversely, MNZ-sensitive strains had high resemblance against reference strains. Because of different mutations in *rdxA*, molecular antibiotic susceptibility testing is not applicable for metronidazole.

LVX was proposed a decade ago based on its role in salvage treatment regimens after the failure of clarithromycin-based treatments [49]. Nevertheless, the frequency of LVX resistance is by all accounts expanding around the world, which might diminish the efficacy LVX-based treatment regimens [50,51]. Our findings showed a high prevalence of primary resistance to LVX (33.1%). Similarly, LVX is not sufficiently effective for inclusion in treatment regimens in Indonesia. Point mutations in the quinolones resistance-determining region (QRDR) of *gyrA* abrogate binding between the antibiotic and the enzyme, resulting in bacterial antibiotic resistance [52]. In the present study we found the predominant mutations at amino acid 87 (Asn to Lys, Tyr, or Ile) and 91 (Asp to Asn, Gly, or Tyr), which have previously been described [18,53,54]. Additionally, these point mutations were present at a high frequency (86.7%) and were associated with high MIC values. Although we found amino acid substitutions at Arg-484 and at Ser-479 in *gyrB* subunits, they had were associated with the *gyrA* mutations in amino acid 87/91, which most likely minimizes the influence of these *gyrB* mutations in Indonesian LVX-resistant strains. Therefore, in Indonesia, screening for *gyrA* mutations could be adequate for recognizing LVX-resistant strains.

The number of *H*. *pylori* strains demonstrating triple or quadruple resistance in this study appeared to be a serious challenge in the fight against infections and a hindrance to the success of eradication regimens. Java and Sumatera Island could be two locations with a higher risk for *H*. *pylori* treatment failure in Indonesia due to the associated high antibiotic resistance type area. Several regions of Indonesia are associated with a high prevalence of *H*. *pylori* infections; increased resistance to the antibiotics used to treat this bacterium might result in increased recurrence rates. It is therefore important to perform susceptibility-guided re-treatment using a case-by-case approach, if available, in patients demonstrating initial treatment failure. Recently, a high accuracy DNA strip genotyping test was developed combining PCR and hybridization that permits the molecular identification of mutations in *gyrA* and *23S rRNA* within 6 h [55]. Our genotypic resistance results are vital to guide follow-up treatment protocols after first-line regimens fail.

The number of samples in this study was relatively low, which certainly suggests the major limitations of this study. We considered that a larger sample size among region is necessary to elucidate the prevalence of *H*. *pylori* antibiotic resistance in Indonesia. The current study is a pilot study for a larger survey and we are now continuing the similar surveys to that performed in this study, to increase sample numbers and expand geographically to other islands. In addition, we only determined the presence of well-known genetic mutations associated with antibiotic resistance. *H*. *pylori* contains approximately 1,600 genes, and it is likely that only a fraction of genomic changes that are related to drug resistance have been identified. Next-generation sequencing technology is beneficial in that it can yield enormous numbers of DNA sequences in less time and at lower cost, which could be used to clarify the evolution and pathogenicity of *H*. *pylori*. To guide antibiotic regimens in Indonesia, the locations were perhaps more important than the ethnicities of the patients. Most antibiotic resistance is related to local antibiotic consumption [56]. Moreover, such resistance is primarily due to the *H*. *pylori* genotype, rather than the human genotype.

## Conclusions

The rates of resistance to MNZ and LVX were high in Indonesia, which implies that MNZ-, and LVX-based triple therapies are not valuable for first-line treatment of *H*. *pylori* in Indonesia. In general, we revealed a low prevalence of CAM, AMX, and TCN resistance. Nevertheless, individuals of several ethnicities were shown to be associated with a high prevalence of CAM, TC, and MNZ resistance. In this manner, CAM- or MNZ-based triple therapy should be used with caution or should be demographic-based in some regions of Indonesia. National epidemiological surveillance of resistance rates is required to further determine optimal treatment strategies in Indonesia.
